# Two Novel *Bacillus* Strains (*subtilis* and *simplex* Species) with Promising Potential for the Biocontrol of *Zymoseptoria tritici*, the Causal Agent of Septoria Tritici Blotch of Wheat

**DOI:** 10.1155/2021/6611657

**Published:** 2021-05-28

**Authors:** Nora Allioui, Fatma Driss, Hanen Dhouib, Lobna Jlail, Slim Tounsi, Olfa Frikha-Gargouri

**Affiliations:** ^1^Department of Ecology and Environmental Engineering, Faculty of Nature and Life Sciences and Earth and Universe Sciences, University of May 8th, 1945 Guelma, Algeria; ^2^Laboratory of Biopesticides, Centre of Biotechnology of Sax, University of Sfax, P.O. Box. “1177”, 3018 Sfax, Tunisia; ^3^Analytical Services Provider Unit, Centre of Biotechnology of Sfax, University of Sfax, P.O. Box. “1177”, 3018 Sfax, Tunisia

## Abstract

Two novel Algerian field-collected isolates were selected for their antifungal activity against *Zymoseptoria tritici* (teleomorph *Mycosphaerella graminicola*). The novel strains, termed Alg.24B1 and Alg.24B2, were identified as *Bacillus subtilis* and *Bacillus simplex* since their respective nucleotide sequences of the 16S rRNA gene were 100% and 99.93% identical to those of *B. subtilis* and *B. simplex*, respectively. The antifungal activities of Alg.24B1 and Alg.24B2 were evaluated by the well diffusion method and compared to those of other *Bacillus* species. The maximum activity was obtained after two days of confrontation of the bacterial strain supernatants with the fungus for Alg.24B1 and three days for Alg.24B2. Furthermore, the metabolites responsible for the antifungal activity of both strains were detected by the investigation of either gene presence (PCR) or molecule production (activity detection of lytic enzymes and HPLC detection of lipopeptides). Overall, this study showed that in addition to their ability to produce lytic enzymes (protease and *β*-glucanase), both strains coproduce three types of lipopeptides *viz.* surfactin, iturin, and fengycin. Thus, the biofungicide activity of both strains may be a result of a combination of different mechanisms. Therefore, they had a great potential to be used as biocontrol agents to effectively manage septoria tritici blotch of wheat (STB).

## 1. Introduction

In Algeria, wheat is the most important crop, and it is the principal consumed food, but national wheat production is very low and does not satisfy the needs of the population. Thereby, the country relies always on imports, which are increasing gradually from one year to the other. Several factors are involved in limiting wheat yields, notably, climate change, diseases, and pests, but fungal foliar diseases pose a real threat. Septoria leaf blotch of wheat (STB) caused by the heterothallic ascomycete fungus *Zymoseptoria tritici* (Desm.), (teleomorph *Mycosphaerella graminicola* (Fuckel) J. Schröt., in Cohn) [1], is one of the most devastating foliar diseases of wheat in Algeria and throughout the world. Serious epidemics can reduce the yield by 35 to 50% [2, 3] via the effects of the disease on yield components and on the grain quality, which can highly decrease in infected plants [4]. In Algeria, during 2010-2012, 80% of 160 prospected fields of durum and bread wheat in 11 localities (East, center, and west of the country), representing the major wheat-producing areas, presented the STB disease, and symptoms have reached the flag leaves of plants [5].

Different chemically synthesized fungicides are commonly used to control *Z. tritici*, but the pathogen has been reported to have great genetic flexibility. Consequently, fungicide-resistant strains were detected in several countries worldwide, including Algeria [6, 7]. Thus, because pesticide use becomes less socially and ecologically acceptable [8], the development of biological methods of crop protection, like the use of beneficial microorganisms (biopesticides), will be a benefit and one of the most promising methods for more rational and safe crop management practices [9]. Biopesticides are important for reducing the risk of resistance to chemical pesticides [10], and the majority of them are biodegradable and less toxic to humans [11] and decompose faster, resulting in lower exposure [12, 13].

Many microorganisms are reported as biocontrol agents and show the potential to control plant pathogens [14]. *Bacillus* species including *B. subtilis*, *B. licheniformis*, *B. pumilus*, *B. amyloliquefaciens*, *B. cereus*, *B. mycoides*, and *B. thuringiensis* are mostly exploited as biopesticides [15, 16] and are of great agriculture importance. These species are known to suppress the growth of several fungal pathogens such as *Rhizoctonia*, *Fusarium*, *Sclerotinia*, *Sclerotium*, *Gaeummanomyces*, *Nectria*, *Pythium*, *Phytophthora*, and *Verticillium* [17, 18]. The first attempts to suppress *Z. tritici* were based on the use of bacteria like *Pseudomonas* [19] and fungi such as *Trichoderma harzianum* and *Gliocladium roseum* [20]. Later on, the potential of the genus *Bacillus* has been mostly investigated. For instance, the potential of *B. megaterium* and *B. subtilis* for the biocontrol of STB on wheat was reported by Kildea et al. [21] and Mejri et al. [22], respectively.

*Bacillus* species are a source of bioactive molecules potentially inhibitory for phytopathogen growth, among which are lipopeptides [23]. Chung et al. [24] reported that the isolate *B. subtilis* ME488 suppressed the growth of 39 of 42 plant pathogens tested. The main documented lipopeptides from *Bacillus* species are iturin [25], surfactin [26], fengycin [27], and kurstakin [28].

Moreover, *Bacillus* spp. produces a range of other metabolites including cell wall-degrading enzymes such as chitinases, *β*-glucanases, and proteases [29, 30]. The number of antibiotics produced by the bacilli class, including the antifungal ones, was approaching 167, being 66 derived from *B. subtilis*, 23 from *B. brevis*, and the remaining antibiotic peptides are produced by other species of *Bacillus* [17].

This work aimed to identify *Bacillus* species endowed with antifungal activity against *Z. tritici*. The compounds responsible for the suppression of STB produced by two novel field-collected *Bacillus* strains were further assessed. Thus, the ability to produce antifungal metabolites (enzymes and lipopeptides) by both isolates was studied. The exploitation of their antifungal activity for their subsequent use to control the disease was discussed.

## 2. Materials and Methods

### 2.1. Biological Material

#### 2.1.1. Fungal Material

Leaves showing symptoms of STB caused by *Z. tritici* (teleomorph *Mycosphaerella graminicola*) were taken from a naturally infected bread wheat field in Guelma (North-East of Algeria), in 2017. Isolation of the fungus was conducted according to the protocol described by Siah et al. [6]. Before use, *Z. tritici* single-spore isolates were grown on Potato Dextrose Agar (PDA, Oxide Ltd., UK) medium for 2 weeks, and then, the fungus was subcultured on PDA and grown at 20°C for 7 days. The suspension was prepared by scraping the surface of the culture in a 0.2% Tween 20 sterile distilled water solution and filtered through sterile cheesecloth before quantification using a Malassez counting chamber. Spore concentration was adjusted to 1 × 10^6^ ml^−1^. The latter suspension preparation method was used to prepare spore suspensions (at the same concentration) from *Fusarium oxysporum*, *Fusarium gramineaurum*, *Botrytis cinerea*, *Aspergillus niger*, and *Alternaria alternata* belonging to the fungal collection of the “Centre of Biotechnology of Sfax” (CBS), Tunisia.

#### 2.1.2. Bacterial Material

Five strains of *Bacillus* sp. were used in this study. V26, C2, and BUPM255 belong to the bacterial collection of CBS, Tunisia. V26 is a *B. subtilis* strain that has a broad antifungal spectrum, including *Fusarium oxysporum*, *Fusarium solani*, *Fusarium gramineaurum*, *Fusarium sambucinum*, *Botrytis cinerea*, and *Rhizoctonia solani* [31–33]. BUPM255 is a *B. thuringiensis* strain active against A*spergillus niger*, *Rhizopus nigricans*, *Fusarium oxysporum*, and *Rhizopus oryzae* [34, 35]. C2 is a *B. amyloliquefaciens* strain that exhibits antifungal activity against *Verticillium dahliae* [36]. Alg.24B1 and Alg.24B2 are isolated from an Algerian soil sample taken from the rhizosphere of wheat plants in Guelma. They were selected among a collection of isolates sampled and tested for their antagonist activity towards *Z. tritici*. Strains were grown at 30°C using LB medium.

### 2.2. Identification of Novel Strains

#### 2.2.1. Classical Taxonomy

The novel isolates Alg.24B1 and Alg.24B2 were identified based on the classical taxonomy criteria such as Gram coloration, oxygen dependence for growth, motility, shape, sporulation, and morphological characteristics of the colonies on LB medium.

#### 2.2.2. Molecular Identification

The molecular methodology was carried out by 16S rDNA sequencing. PCR amplification was carried out using the universal primers Fd1 (5′-AGAGTTTGATCCTGGCTCAG-3′) and Rd1 (5′-AAGGAGGTGATCCAGCC-3′), designed from the conserved zones within the rRNA operon of *E. coli* [37]. The genomic DNA of the isolates Alg.24B1 and Alg.24B2, extracted from the LB-cultured bacterial cells by standard protocols [38], was used as a template for PCR amplification. PCR products were purified using EZ-10 spin column DNA Gel Extraction Kit (BIO BASIC INC., Canada). Thermal cycler conditions consisted of an initial denaturation at 94°C for 2 min followed by 30 cycles; each one composed of denaturation at 94°C for 30 s, annealing at 53°C for 1 min, and extension at 72°C for 2 min. The 1.5 kb amplicons were purified from the agarose gel and sequenced in an automatic sequencer (Avant Genetic analyzer, 3100 model, Applied Biosystems, CA, USA). Homology search was performed using the BLAST algorithm [39] within the NCBI database (http://www.ncbi.nlm.nih.gov/BLAST/). Accession numbers obtained from GenBank for deposited partial nucleotide sequence for 16S ribosomal RNA genes were MW692842.1 and MW692843.1 for Alg.24B1 and Alg.24B2, respectively.

### 2.3. Antifungal Activity Assays

The antifungal activity of cell-free filtrate was evaluated by adaptation of the well diffusion method [34]. In brief, a fungal spore suspension (1 × 10^6^ ml^−1^ in 0.2% Tween) was spread on the surface of PDA and incubated at 20°C in the dark for 24 h. After 48 h culturing time in LB medium, 100 *μ*l of the cell-free culture supernatant were placed in the center of the wells punched in the PDA plates. 100 *μ*l of LB were used as a negative control. Plates were incubated as before and the growth inhibition zones around the bacterial supernatant were evaluated. The evaluation of the antifungal activity against *F. oxysporum*, *F. gramineaurum*, *B. cinerea*, *A. niger*, and *A. alternata* was done by checking the appearance of growth inhibition zones. However, in the case of *Z. tritici*, the diameters of the growth inhibition zones were daily measured (cm) for five days. Besides, the specific activity (UA ml^−1^) was calculated for both strains, Alg.24B1 and Alg.24B2, using the serial dilution testing [40]. One arbitrary unit (AU) of the antifungal agent was defined as the amount of cell-free culture supernatant sufficient to give a zone of inhibition around the well and calculated as the reciprocal of the highest dilution factor of the sample. All experiments were conducted three times.

### 2.4. Effect of Proteinase K on Antifungal Activity

Cell-free supernatants with antifungal activity against *Z. tritici* were incubated at 37°C for 1 h with proteinase K (1 mg ml^−1^). The enzyme inactivation was performed by boiling for 10 min. Supernatants without the addition of proteinase K served as a negative control. All samples were then tested for their antifungal activity against *Z. tritici*.

### 2.5. Lytic Enzyme Production

Protease, *β*-glucanase, and chitinase activities were evaluated by culturing the bacterial strains on skim milk [41], barley flour [42], and colloidal chitin agar plates [34], respectively. A clear zone around the colony after two days of incubation at 30°C indicated the enzymatic degradation. The plates were flooded with Congo red solution (0.1%) for 15 min. The appearance of halo zones was considered a positive response for lytic activity.

### 2.6. PCR Detection of Lipopeptide Biosynthetic Genes

Primers used in the PCR amplifications are listed in Table 1. PCR amplifications were conducted in a 50 *μ*l reaction mixture containing 50 ng of template DNA, 10 *μ*l of 5x PCR buffer, 4 *μ*l of 25 mmol l^−1^ MgCl_2_, 5 *μ*l of dNTP mix (0.2 mmol l^−1^), 5 *μ*l of each forward and reverse primer (10 mmol l^−1^), and 2 U of Taq DNA polymerase (GoTaq, Promega, Madison, WI, USA). Thermal cycler conditions consisted of an initial denaturation step at 95°C for 5 min, followed by 30 cycles of denaturation at 95°C for 1 min, primer annealing at the appropriate temperature for 1 min, and extension at 72°C for 1.5 min followed by a final extension step at 72°C for 7 min. Bacillus strain 32a, which was previously identified as *B. amyloliquefaciens* by Ben Abdallah et al. [43], was used as a positive control.

### 2.7. Isolation, Identification, and Evaluation of Lipopeptides Using HPLC Analysis

The bacterial strain cultures were carried out at 30°C, in 50 ml LB, with an initial OD of 0.1 and at a rotation of 200 rpm. Cultures were harvested by centrifugation at 12,000 rpm for 15 min and then filtrated through a 0.22 *μ*m cut-off filter. Cell-free cultures were purified on a C18 SPE column. The eluted fractions were concentrated *in vacuo* and then analyzed by HPLC using as mobile phase Milli-Q water and acetonitrile. The elution was performed using a gradient of 40–100% acetonitrile (56 min) at a flow rate of 0.6 ml min^−1^. UV detection used a wavelength of 214 nm. The elution times for the obtained groups of peaks were compared to those observed for commercial standards (Lipofabrik, Villeneuve d'Ascq, France). Lipopeptide yields were calculated by HPLC peak area based on values obtained for pure products [45]. Experiments were carried twice.

### 2.8. Statistical Analysis

The data were subjected to analysis using the Statistical Package for the Social Sciences (SPSS Statistics17.0; SPSS Inc., Chicago, IL, United States). The mean values among the measurements were compared using Duncan's multiple range test at the 5% level of significance (*p* = 0.05).

## 3. Results and Discussion

A collection of Algerian bacteria originating from the rhizosphere of wheat plants were screened for antifungal activity against *Z. tritici* by plate assays using the confrontation method. Two bacterial isolates were selected as they exhibited the highest antifungal activity against *Z. tritici* amongst the bacteria of the collection. The selected isolates were termed Alg.24B1 and Alg.24B2. They were first identified, and then, their activities were evaluated by comparison with other strains. Finally, their biofungicide compounds were investigated.

### 3.1. Characterization and Identification of the Novel Isolates Alg.24B1 and Alg.24B2

#### 3.1.1. Spectrum of Antifungal Activity

In addition to *Z. tritici*, the antifungal activities of both isolates were tested against several phytopathogenic fungi, especially those causing cereal diseases *viz. F. oxysporum* and *F. gramineaurum* or crop diseases in general, namely, *B. cinerea*, *A. niger*, and *A. alternata*. Isolate Alg.24B1 showed a larger spectrum of activity than Alg.24B2. Indeed, Alg.24B1 showed remarkable activity against all tested fungi, while the activity was lower for Alg.24B2 and was restricted to *A. niger* and *F. gramineaurum*.

#### 3.1.2. Classical Identification

Classical taxonomic findings showed that the newly isolated bacteria Alg.24B1 and Alg.24B2 are Gram-positive, aerobic, motile, rod-shaped, and spore-forming bacteria. LB plating showed that Alg.24B1-colonies have an irregular shape, dry, flat, and irregular with serrated margins, but Alg.24B2-colonies are creamy, glossy with irregular margins, slightly raised, and umbonate. However, the diversity of the growth patterns of *B. subtilis* colonies can be observed, and it is related to the availability of nutrients in the agar plate [46]. Caulier et al. [47] reported that the genus *Bacillus* comprises 377 species (last updated in January 2019) of Gram-positive, rod-shaped bacteria. Their ability to form endospores, their diversity in physiological properties, as well as their capacity to produce numerous antimicrobial compounds, favor their ubiquitous distribution in soil, aquatic environments, food, and gut microbiota of arthropods and mammals.

#### 3.1.3. Molecular Identification

Alg.24B1 and Alg.24B2 were also identified by a molecular approach. Thereby, the genomic DNA of both isolates was used as a template to amplify PCR-fragments coding for the 16S rRNA. The DNA fragments were purified and sequenced. Considering the length of sequence overlap (Table 2), DNA similarity searches against bacterial databases revealed that the 16S rRNA sequence of Alg.24B1 was 100% identical to *B. subtilis*, while that of Alg.24B2 was more than 99.93% identical to *B. simplex*. These results are consistent with those obtained by the classical identification. Thus, Alg.24B1 and Alg.24B2 were identified as *B. subtili*s and *B. simplex*, respectively.

The ability of *B. subtilis* to suppress STB was recently documented, and the compounds involved in the activity against *Z. tritici* were studied [22]. However, to the best of our knowledge, the antifungal activity of *B. simplex* has never been reported against *Z. tritici*.

### 3.2. In Vitro Evaluation of the Effect on Z. Tritici Growth

**Figure 1:**
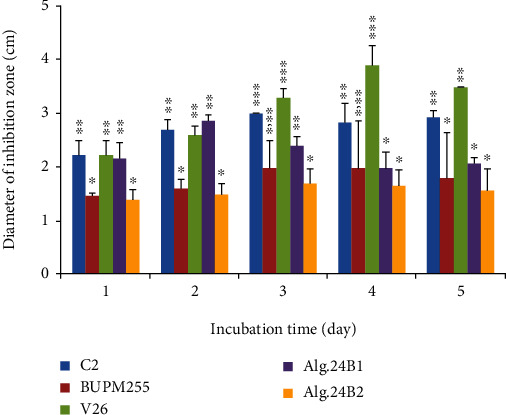
Effect of the *Bacillus* sp. strains on the *in vitro* growth of *Z. tritici*. Values were reported as means ± SD of three measurements. Vertical bars represent standard errors of the means. “^∗^”, “^∗∗^”, and “^∗∗∗^” show statistically significant differences between the tested *Bacillus* species each day separately. *p* values less than 0.05 were considered statistically significant.

Antimicrobial assays using cell-free supernatants showed specific activities against *Z. tritici* of 320 and 54 UA ml^−1^ and maximum inhibitory activities at two and three days of confrontation for Alg.24B1 and Alg.24B2, respectively. Additionally, the antifungal activity of these two newly identified strains against *Z. tritici* was compared with those of *Bacillus* strains of different species endowed with antifungal activities listed in the materials and methods section, namely, *B. amyloliquefaciens* (C2), *B. subtilis* (V26), and *B. thuringiensis* (BUPM255) (see Figure S1 in the Supplementary Materials for the illustration of the confrontation test). Equal inhibition zone diameter lengths were obtained after one and two days of confrontation with *Z. tritici* for Alg.24B1 (*B. subtilis*), C2 (*B. amyloliquefaciens*), and V26 (*B. subtilis*), on the one hand, and for Alg.24B2 (*B. simplex*) and BUPM255 (*B. thuringiensis*) on the other hand. The Alg.24B1 group was more effective than that including Alg.24B2 since it showed an earlier and higher maximum of activity (Figure 1). Compared to V26, which presented the same species as Alg.24B1 (*B. subtilis*), the latter reached the maximum of activity after only two days of confrontation, while V26 reached it after four days, even though with a larger diameter than that of Alg.24B1. This could be an essential asset for a low-cost mass production bioprocess since an early production of metabolites is always desired.

In a similar context, Schwartz et al. [48] have tested the antifungal activity of these two *Bacillus* subspecies: *B. subtilis* 30VD-1 and *B. simplex* 30N-5 against *Fusarium oxysporum* and the pea pathogen *Nectria haematococca*. They have shown that *B. subtilis* 30VD-1 was a robust fungal antagonist as it can reduce the fungal growth by 50% or more after 6 days of cocultivation. However, *B. simplex* 30N-5 was not an as effective antagonistic agent as strain *B. subtilis* 30VD-1, in part because of its reduced growth in most cocultivation media. *B. simplex* 30N-5 limited fungal radial expansion to about one-half the level of that of *B. subtilis* 30VD-1. Thus, the authors concluded that both strains have the potential for use in biocontrol. Likewise, we believe that our strains have the potential to be used for biocontrol. Hence, in the following section, we investigated the metabolites responsible for their antifungal activities.

### 3.3. Detection of Metabolites Potentially Responsible for the Antifungal Activity

Potential contributing factors to the biofungicide activity of both strains were analyzed by the investigation of either gene presence or biomolecule production.

#### 3.3.1. Investigation of Enzymatic Activities

**Figure 2:**
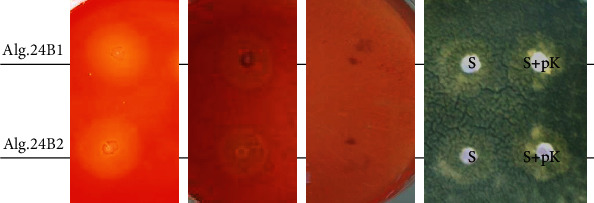
Detection of enzymatic activities in strains Alg.24B1 and Alg.24B2. (a) Protease activity. (b) *β*-glucanase activity. (c) Chitinase activity. (d) Effect of proteinase K on the antifungal activity of both strains: S: supernatant; S+pK: supernatant treated by proteinase K.

Three types of enzymatic activities were investigated *viz.* chitinase, protease, and *β*-glucanase activities, since the corresponding lytic enzymes are known to be cell-wall degrading hydrolases and have thus an essential role in the antifungal process [49]. Our results showed that both strains exhibited protease (Figure 2(a)) and *β*-glucanase (Figure 2(b)) activities, but no chitinase activity was detected at the testing conditions (Figure 2(c)). This feature is essential for the selection of these bacterial isolates to be used as biological control agents. Thus, further investigations should be carried out to characterize these enzymes.

Furthermore, the sensitivity of the respective cell-free supernatants to proteinase K was tested to determine if the antifungal activity produced by both strains against *Z. tritici* is only enzymatic. A slight reduction of the growth inhibition of *Z. tritici* in the presence of the supernatants treated with proteinase K was observed compared to the untreated ones (Figure 2(d)). This suggests that, in addition to the bioactive compounds of protein nature, both studied *Bacillus* strains secrete other compounds which are not of protein nature such as lipopeptides which antifungal activities are increasingly studied. Therefore, we focused on the ability of our isolates to produce lipopeptides.

#### 3.3.2. Investigation of Lipopeptides

Last years, the antifungal activity of lipopeptides has been increasingly investigated in *Bacillus* species, especially in *B. subtilis*. Indeed, iturin produced by *B. subtilis* strains shows a broad antifungal spectrum, making it an ideal potential biological control agent [50]. Besides, surfactin A and its homologs have recently been reported to possess antifungal activity [51]. Also, fengycins produced by several *B. subtilis* strains are known to develop antifungal activity against filamentous fungi. They are responsible for membrane leakage and thus for the bioactivity of the bacterium against fungi [52]. The potential of these lipopeptides from *B. subtilis* [iturin (mycosubtilin), surfactin, and fengycin] has been assessed to suppress STB. A significant reduction in disease severity was found for mycosubtilin [22]. Furthermore, lipopeptides are considered natural control products that exhibit much less ecotoxicity than chemical fungicides. Therefore, we investigated lipopeptides from both isolates.

#### 3.3.3. Molecular Investigation

**Figure 3:**
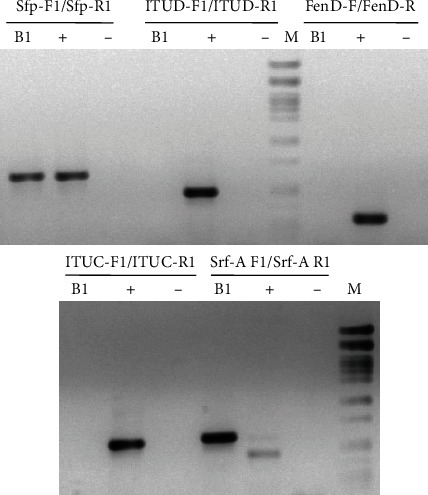
PCR results for Alg.24B1 strain (B1). (+) positive control, (-) negative control, (M) DNA marker: Lambda/*Pst*I.

To determine whether the newly identified strains have the potential to produce different types of lipopeptides, especially those with reported antifungal activities, namely, surfactin, iturin, and fengycin; nonribosomal peptide synthetase genes were investigated. This method was chosen since these genes were reported to be used as markers for the identification and selection of novel biocontrol agents from environmental samples [53, 54]. Thus, the PCR method using appropriate primers was employed, first for the detection of surfactin, iturin, and fengycin for both isolates. The ITUD-F1/R1 primer pair was used to simultaneously screen for *bamD*, *ituD*, and *fenF*, which are conserved genes that encode for malonyl-CoA transacylases involved in the biosynthesis of the lipopeptides bacillomycin D, iturin, and mycosubtilin, respectively [55]. The other primer pairs were specific for genes involved in the biosynthesis of an individual antibiotic. Amplicons of the expected sizes were obtained only with surfactin for Alg.24B1 (Figure 3). However, no amplicons were obtained for Alg.24B2 (data not shown).

Mora et al. [44] have examined the presence of the antimicrobial peptide (AMP) biosynthetic genes *srfAA* (surfactin), *bacA* (bacylisin), *fenD* (fengycin), *bmyB* (bacyllomicin), *spaS* (subtilin), and *ituC* (iturin) in 184 isolates of *Bacillus* strains. They found that most strains had between two and four AMP genes. The most frequent AMP gene markers were *srfAA*, *bacA*, *bmyB*, and *fenD*, and the most frequent genotypes were *srfAA-bacA-bmyB* and *srfAA-bacA-bmyB-fenD*. It was suggested that “the dominance of these particular genes in *Bacillus* strains associated with plants reinforces the competitive role of surfactin, bacyllomicin, fengycin, and bacilysin in the fitness of strains in natural environments.”

**Figure 4:**
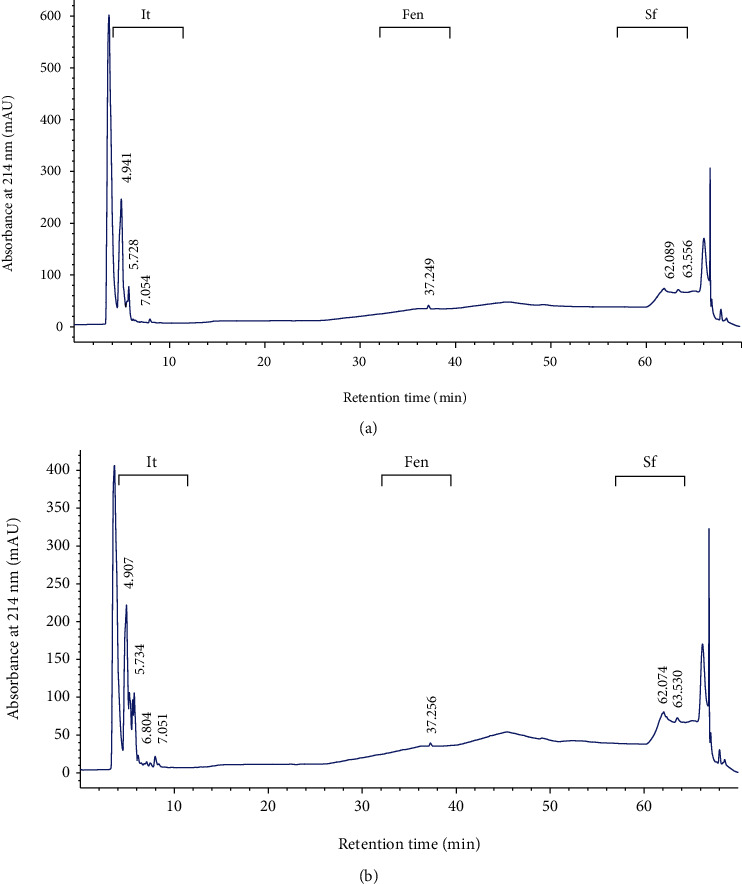
Chromatogram of the HPLC analysis of purified extracts from Alg.24B1 (a) and Alg.24B2 (b). Peaks corresponding to iturin (It), fengycin (Fen), and surfactin (Sf) are indicated.

*(1) Biochemical Investigation*. In a second step, the lipopeptides from both strains were partially purified by SPE on a C18 column and then chromatographically separated by HPLC in reverse phase on a C18 column (Figure 4). Data analysis was carried out by comparing crop profiles with standards. For the strain Alg.24B1, the iturin family was detected at three retention times of 4.941, 5.728, and 7.064, the fengycin family was observed at one retention time of 37.249, and the surfactin one was obtained at two retention times of 62.089 and 63.556 (Figure 4(a)). A similar profile was obtained for the strain Alg.24B2: four retention times of 4.907, 5.734, 6.804, and 7.061 for the iturin family, one retention time of 37.256 for the fengycin family, and two retention times of 62.074 and 63.530 for the surfactin one (Figure 4(b)).

No correlation between genes and products of the two potent strains was observed. Indeed, although iturin and fengycin were produced by Alg.24B1, no PCR amplification of the corresponding genes was obtained. Only the surfactin family was detected in this strain by both molecular (PCR) and biochemical (HPLC) methods. Moreover, even though no PCR amplification was obtained for Alg.24B2, all lipopeptide families were detected in the HPLC retention profile of the strain. Similar results dealing with a low correlation between PCR and HPLC outcomes were reported. For example, Mora et al. [54] faced this problem while studying the *bm*yB and *srf*AA genes and their corresponding products. Besides, Frikha-Gargouri et al. [56] have obtained the same trouble while studying these same genes. The main conclusions advanced on this point were that this low correlation is attributed to mutations [56] and the differential production of lipopeptides according to the growth medium used [57, 58].

Both strains could be considered amongst the few strains that produce more than one type of lipopeptide. According to Wu et al. [51], most *Bacillus* sp. can produce one type of lipopeptides and a few can produce two or three types of lipopeptides. Moreover, Sandrin et al. [59] have tested 13 strains of *B. subtilis* for the coproduction of surfactin and iturin. They found that only one strain (*B. subtilis* S499) produced both lipopeptides with a high yield. Pyoung et al. [60] reported that *B. subtilis* CMB32 produced three types of antifungal lipopeptides (Iturin A, fengycin, and surfactin A).

The mode of action of lipopeptides along the antifungal process is not clearly established. It is the subject of several investigations. Indeed, Le Mire et al. [61] showed that, by *in vivo* test, surfactin from the strain *B. amyloliquefaciens* S499 protected wheat by 70% against *Z. tritici*, but *in vitro* biocidal assays revealed no antifungal activity of surfactin towards the pathogen. They concluded that surfactin significantly induced wheat natural defense by stimulating both salicylic acid- and jasmonic acid-dependent signaling pathways. Mejri et al. [22] suggested that iturin has an antifungal property, but surfactin and fengycin had no direct activity on the pathogen and act on wheat against *Z. tritici* as resistance inducers rather than as biofungicides. Moreover, the biological activities of lipopeptides are closely related to the sequence of amino acid residues, the cyclization of the peptide, and the length and branching of the fatty acid chain [51].

The evaluation of the lipopeptide yields from both strains was carried out by HPLC (Table 3). A large amount of iturin was produced by Alg.24B1 (3120 mg l^−1^). However, this quantity is not optimal and an optimized medium could be used for higher yields. Indeed, higher quantities were reported for *B. subtilis* strain RB14 which produces 4450 mg l^−1^ and 5050 mg l^−1^ of iturin by submerged fermentation and by biofilm fermentation using maltose and fish protein as sources of carbon and nitrogen, respectively [62]. Habe et al. [63] reported that the culturing conditions such as the culture medium, aeration rate, and agitation speed are crucial factors in the metabolite production process in general, and especially that of iturin from *B. subtilis*. The surfactin yield produced by Alg.24B1 was 128.17 mg l^−1^, which is similar to that reported by Sandrin et al. [59], and Hsieh et al. [64] (110 mg l^−1^ and 125,6 mg l^−1^, respectively). Mohammadipour et al. [65] have reported that *B. subtilis* produces from 55 to 1610 mg l^−1^ of surfactin. According to Zhi et al. [66], *Bacillus* isolates generate limited amounts of surfactin (<10% of their biomass), which functions as an antibiotic or as a signaling molecule in inter-/intraspecific interactions. However, overproduction of surfactin by *B. amyloliquefaciens* MT45 was observed at a titer of 2930 mg l^−1^, which is equivalent to half of the maximum biomass. The amounts of fengycin produced by both strains are the lowest compared with those of iturin and surfactin (Table 3), which is in agreement with the results reported by Chowdhury et al. [67] for *B. amyloliquefaciens*. The amount of fengycin could be increased when special culture conditions are applied. Indeed, Coutte et al. [68] reported that 242 mg l^−1^ of fengycin were produced by *B. subtilis* ATCC 21332 with special aeration by a hollow fiber membrane air-liquid contactor (polypropylene). Likewise, an amount of 3550 mg l^−1^ was obtained at optimal medium composition by *B. subtilis* F29-3 [69].

Even though Alg.24B2 produces the three types of lipopeptides, the production yields were lower than those of Alg.24B1. This could explain why Alg.24B1 has a better and wider spectrum of activity than Alg.24B2. Consequently, the degree and nature of activity are widely dependent on the concentration of lipopeptides produced by the isolate [70].

Taken together, our results showed that, on the one hand, the coproduction of iturin, fengycin, and surfactin by our novel isolates Alg.24B1 and Alg.24B2 could be a good asset for their use as biological control agents. It was reported that when different families of lipopeptides are coproduced, their interaction can become synergistic and enhances each of their respective activities [71]. On another hand, the simultaneous production of lipopeptides and cell wall degrading enzymes *viz. β*-glucanase and protease could be a second asset for the exploitation of these potentialities for *Z. tritici* biocontrol. Indeed, the theory speculating that more than one mechanism could be act synergistically to suppress the disease in some specific plant-pathogen systems has been given [72].

## 4. Conclusion

The present study demonstrated the potential of two novel strains Alg.24B1 (*B. subtilis*) and Alg.24B2 (*B. simplex*) to be used as biocontrol agents on wheat against *Z. tritici*. More interest should be given to Alg.24B1 since it exhibits a wider spectrum of antifungal activity and higher yields of three types of lipopeptides (iturin, surfactin, and fengycin). The simultaneous production of three types of lipopeptides added to the production of lytic enzymes reported to be antifungal metabolites could be a good asset to the use of our new isolates for the suppression of STB. Therefore, further *in vivo* tests should be carried out to evaluate the potential of the isolates to be used as biocontrol. Besides, studies are to be undertaken for the identification and characterization of the produced lipopeptides from both strains since the biological activities of lipopeptides strongly depend on their structures. Finally, we believe that optimized culture conditions and formulations of these novel isolates, especially Alg.24B1, should be developed for effective biocontrol of *Z. tritici*, and eventually other phytopathogenic fungi.

## Figures and Tables

**Table 1:** Primers used for PCR for gene content detection.

Lipopeptide	Gene(s)	Primers	Sequences (5′—3′)	PCR product size (bp)	Reference
Fengycin	*fenD*	FEND-F	GGCCCGTTCTCTAAATCCAT	269	[44]
		FEND-R	GTCATGCTGACGAGAGCAAA		

Iturin	*ituD*	ITUD-F1	TTGAAYGTCAGYGCSCCTTT	482	[24]
		ITUD-R1	TGCGMAAATAATGGSGTCGT		
	*ituC*	ITUC-F1	CCCCCTCGGTCAAGTGAATA	594	
		ITUC-R1	TTGGTTAAGCCCTGATGCTC		

Surfactin	*sfP*	SFP-F1	ATGAAGATTTACGGAATTTA	675	[24]
		SFP-R1	TTATAAAAGCTCTTCGTACG		
	*srf*	Srf-A F1	AGAGCACATTGAGCGTTACAA	626	[24]
		Srf-A R1	CAGCATCTCGTTCAACTTTCAC		

**Table 2:** Identification of bacteria based on 16S rRNA sequence homology.

Isolate	NCBI strain compared to	Length of sequence overlap (bp)	Percentage of homology
Alg.24B1	*Bacillus subtilis* MT645613.1	1309	100%
Alg.24B2	*Bacillus simplex* MF977326.1	1343	99.93%

**Table 3:** Yield evaluation of lipopeptides from Alg.24B1 and Alg.24B2.

Yield (mg l^−1^)	Iturin	Fengycin	Surfactin
Alg.24B1	3120.00	0.42	128.17
Alg.24B2	1400.00	0.17	23.42

## Data Availability

The figures used to support the findings of this study are included within the supplementary information file named “Figure S1.”

## Supplementary Materials

Supplementary Materials Figure S1: An illustration of the antifungal activity against *Z. tritici* by adaptation of the well diffusion method. 100 *μ*l of 48 h-cell-free filtrates of the bacterial strains were used. Photos were taken after two days of confrontation with *Z. tritici*.
