# Supplementary material for: Two Novel Bacillus Strains (subtilis and simplex Species) with Promising Potential for the Biocontrol of Zymoseptoria tritici, the Causal Agent of Septoria Tritici Blotch of Wheat
Source: Biomed Res Int. 2021 May 28;2021:6611657. doi: 10.1155/2021/6611657 (PMC8183297; doi:10.1155/2021/6611657)
Supplement: Supplementary Materials — Figure S1: An illustration of the antifungal activity against Z. tritici by adaptation of the well diffusion method. 100 μl of 48 h-cell-free filtrates of the bacterial strains were used. Photos were taken after two days of confrontation with Z. tritici. [file 6611657.f1.pdf]

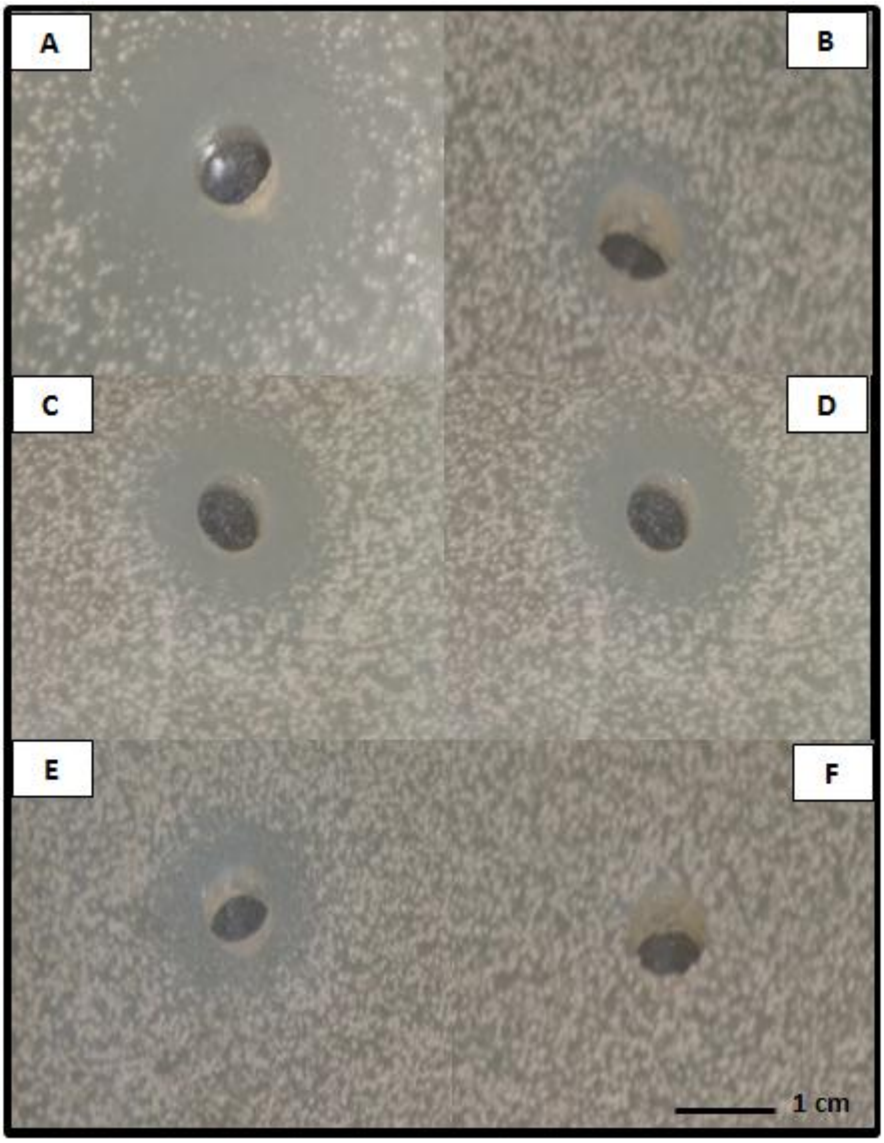

Figure S1: Illustration of the antifungal activity against *Z. tritici* by adaptation of the well diffusion method. 100 µl of 48 h-cell-free filtrates of the bacterial strains were used. Photos were taken after two days of confrontation with *Z. tritici*. A: Alg.24B1, B: Alg.24B2, C: C2, D: V26, E: BUPM255, F: negative control.
